# Selection of Network Parameters in Direct ANN Modeling of Roughness Obtained in FFF Processes

**DOI:** 10.3390/polym17010120

**Published:** 2025-01-06

**Authors:** Irene Buj-Corral, Maurici Sivatte-Adroer, Lourdes Rodero-de-Lamo, Lluís Marco-Almagro

**Affiliations:** 1 Department of Mechanical Engineering, Barcelona School of Industrial Engineering (ETSEIB), Universitat Politècnica de Catalunya, Av. Diagonal, 647, 08028 Barcelona, Spain; 2 Department of Mechanical Engineering, Polytechnic School of Engineering of Vilanova i la Geltrú (EPSEVG), Universitat Politècnica de Catalunya, Av. Víctor Balaguer, 1, 08880 Vilanova i la Geltrú, Spain; maurici.sivatte@upc.edu; 3 Department of Statistics and Operations Research, Barcelona School of Industrial Engineering (ETSEIB), Universitat Politècnica de Catalunya, Av. Diagonal, 647, 08028 Barcelona, Spain; lourdes.rodero@upc.edu (L.R.-d.-L.); lluis.marco@upc.edu (L.M.-A.)

**Keywords:** FFF, FDM, Polylactic acid, artificial neural networks, surface roughness, training algorithm, number of neurons, datasets distribution, multilayer perceptron, backpropagation algorithm

## Abstract

Artificial neural network (ANN) models have been used in the past to model surface roughness in manufacturing processes. Specifically, different parameters influence surface roughness in fused filament fabrication (FFF) processes. In addition, the characteristics of the networks have a direct impact on the performance of the models. In this work, a study about the use of ANN to model surface roughness in FFF processes is presented. The main objective of the paper is discovering how key ANN parameters (specifically, the number of neurons, the training algorithm, and the percentage of training and validation datasets) affect the accuracy of surface roughness predictions. To address this question, 125 3D printing experiments were conducted changing orientation angle, layer height and printing temperature, and measuring average roughness Ra as response. A multilayer perceptron neural network model with backpropagation algorithm was used. The study evaluates the effect of three ANN parameters: (1) number of neurons in the hidden layer (4, 5, 6 or 7), (2) training algorithm (Levenberg–Marquardt, Resilient Backpropagation or Scaled Conjugate Gradient), and (3) data splitting ratios (70%–15%–15% vs. 55%–15%–30%). Mean Absolute Error (MAE) was used as the performance metric. The Resilient Backpropagation algorithm, 7 neurons, and using 55% of training data yielded the best predictive performance, minimizing the MAE. Additionally, the impact of the dataset size on prediction accuracy was analysed. It was observed that the performance of the ANN gets worse as the number of datasets is reduced, emphasizing the importance of having sufficient data. This study will help to select appropriate values for the printing parameters in FFF processes, as well as to define the characteristics of the ANN to be used to model surface roughness.

## 1. Introduction

Polylactic acid (PLA) is a biocompatible material that can be used in medical applications, for example to manufacture surgical models [1]. It can also be employed in tissue engineering, for instance to favor the integration of tissue-engineered bone with natural bone or to produce scaffolds to regenerate epithelial cells [2]. It allows obtaining biodegradable orthopedic devices [3]. Moreover, PLA is a bioresorbable polymer, commonly used for stents and sutures [4].

Fused Filament Fabrication (FFF), also known as Fused Deposition Modeling (FDM), consists of extruding material through a nozzle and depositing it to form a part layer by layer. Main advantages of this process are its easiness of use, its low cost if desktop technologies are employed, and the possibility to use different plastic materials [5]. Some of their drawbacks are its low dimensional accuracy [6,7] and its poor surface roughness in lateral walls [8], because of the ridge pattern which is formed by the beads’ edges.

Post-processing operations are often employed to reduce surface roughness of 3D printed parts. They can either be mechanical, chemical, thermal, or hybrid. For example, the surface peaks can be mechanically cut, pressed, or polished [9]. As for the chemical post-processing of plastic materials, for example acetone vapor is used in acrylonitrile butadiene styrene (ABS) parts [10]. Regarding thermal methods, for instance, laser polishing has been used in PLA parts to improve their surface finish [11]. However, post-processing methods could affect the structure and the mechanical properties of the parts [9]. Moreover, in addition to layer height, other 3D printing conditions can affect surface roughness. For example, for a certain layer height value, roughness increases in inclined walls due to the stair-stepping effect. Thus, print orientation angle has a great influence on roughness [12]. Hooshmand et al. [13] found that both build orientation and layer height have an important effect on surface roughness in FFF processes, with 0° and 0.22 mm, respectively, leading to optimal results. Similarly, Abas et al. [14] found lowest Ra values when build orientation values of 0° were selected, using I-optimal design and definitive screening design (DSD). In addition, printing temperature can affect roughness because it can lead to deformation of the deposited beads [15,16]. Specifically, roughness decreases when temperature increases from 210 °C to 230 °C in thin-walled PLA parts [17].

Artificial Neural Network (ANN) models have several advantages over regression or analytical models, such as their high computation, quick learning, and powerful memory [18]. Neural networks have been used in the past to model roughness in different manufacturing processes like milling [19] or honing [20]. In FFF processes, for example, Shirmohammadi et al. [21] employed ANN and particle swarm algorithm to model roughness in the lateral walls of the parts. They selected five process parameters, namely printing temperature, layer thickness, printing speed, nozzle diameter, and material density. They used a multi-layered perceptron neural network (7-4-1). They reported an error of 4.88%, while a surface response quadratic model with the same data showed an error of 8.75%. Tripathi and Singla [22] used ANN, with layer thickness, infill density, nozzle temperature, wall thickness, bed temperature, material, fan speed, print speed, and infill pattern as variables, and elongation, tensile strength and surface roughness as responses of the model. They used models with four layers.

As for the selection of best parameters of the networks, for example, Ke and Huang [23] combined systematic experimental design with ANN modeling in injection molding processes. They highlight the importance of selecting appropriate quality indices and optimizing neural network parameters to increase accuracy. For example, using the same number of neurons in the hidden layer as in the input layer provided quality training results. In FFF processes, Chinchanikar et al. [24] predicted surface roughness from different parameters, including number of neurons, number of hidden layers, layer height, infill density, nozzle temperature, and print speed. In this case, best results were obtained with two hidden layers and 150 neurons in each layer. However, when modeling FFF processes, there is little information in the literature about the neural network topology to be employed.

The main objective of the paper is discovering how key ANN parameters (specifically, the number of neurons, the training algorithm, and the percentage of training and validation datasets) affect the accuracy of surface roughness predictions in FFF processes. For this purpose, 125 3D printing experiments were conducted, changing print orientation angle, layer height, and printing temperature, and measuring average roughness Ra as response. A multilayer perceptron neural network model with a backpropagation algorithm is used. Finally, a study is performed to assess the effect of dataset size on the performance of the networks.

## 2. Materials and Methods

### 2.1. 3D Printing of the Samples

**Figure 1:**
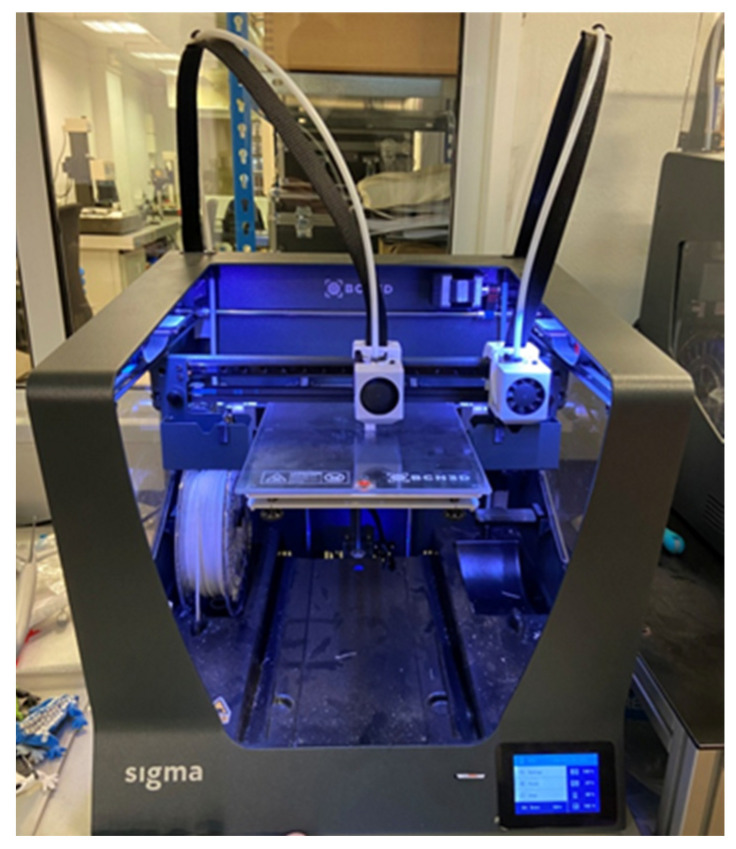
Sigma R19 3D printer.

A Sigma R19 3D printer from BCN3D Technologies (Gavà, Spain) was used to print the samples (Figure 1).

Different cylindrical specimens of diameter 12.7 mm and height 25.4 mm were printed in Polylactic acid (PLA). The 3D printing parameters are presented in Table 1. In the experiments, nozzle diameter, infill ratio, and print speed were fixed, while print orientation angle (OA), layer height (LH), and printing temperature (PT) were varied, according to a 5^3^ full factorial design. A total amount of 125 experiments was carried out (see Table A1 in Appendix A).

Nozzle diameter value of 0.4 mm and print speed value of 40 mm/s are usual in the FFF 3D printing of PLA. A high infill ratio of 80% was considered, so that high mechanical strength will be achieved. Print orientation angle covers the range between 0 and 80°. It excludes 90° (horizontal cylinder), which is a special case that provides low surface roughness values, since the measurement direction is parallel to the printed layers [12]. Layer height covers a wide range of values from 0.05 mm to 0.25 mm, all of them below the nozzle diameter value of 0.4 mm. Printing temperature ranges between 190 °C and 210 °C, which are usual when 3D printing PLA [25]. Similar values were used by Kugunavar et al. [26] for print orientation angle, layer height, and printing temperature, when analyzing roughness with the Taguchi method.

**Figure 2:**
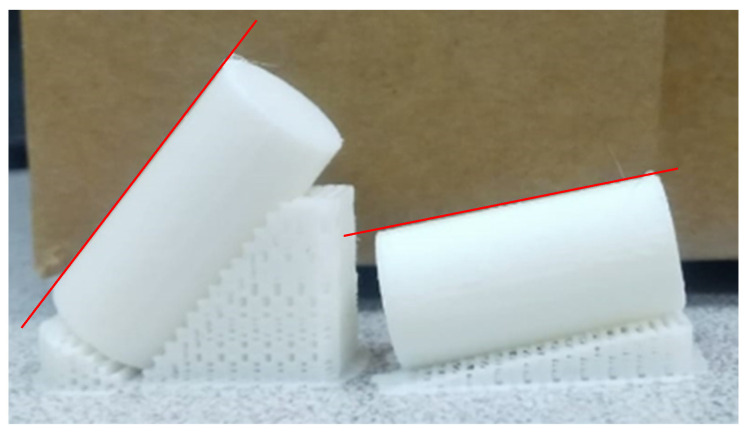
Three-dimensional printed cylinders with print orientation angle 40° (left) and 80° (right).

Figure 2 shows two different specimens obtained with layer inclination 50° (left) and 10° (right), corresponding to print orientation 40° (left) and 80° (right).

### 2.2. Roughness Measurement

Roughness was measured with a Talysurf 2 (Taylor Hobson Ltd., Leicester, UK) contact roughness meter. The samples were placed on V-shaped blocks. A diamond tip was used with tip radius of 2 µm. Resolution of the inductive sensor is 18 nm.

Roughness profiles were measured along a generatrix of the samples (red line in Figure 2), which corresponds to their lateral walls. Specifically, the generatrix was measured that is placed opposite to the printing supports. For print orientation angle of 0° (vertical cylinder), no printing supports are required and a random generatrix of the part is measured. In this case, the measurement direction is perpendicular to the printing layers.

A Gaussian filter was considered with a cut-off length of 0.8 mm and an evaluation length of 4 mm. The average roughness parameter Ra was determined from the roughness profiles.

### 2.3. Neural Networks

**Figure 3:**
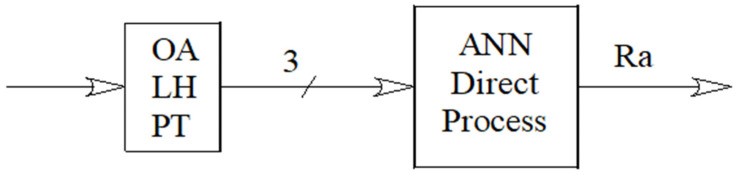
Schematic diagram of the ANN employed.

Figure 3 shows a schematic diagram of the network employed, in which the direct problem is addressed. It has three inputs, namely print orientation angle (OA), layer height (LH), and printing temperature (PT), and one output, average roughness Ra.

**Figure 4:**
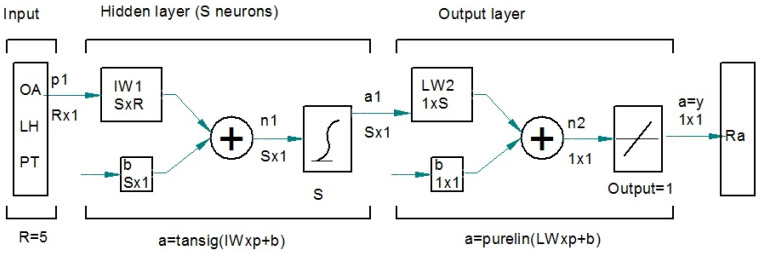
Structure of the ANN employed.

The detailed structure of the network is provided in Figure 4.

The ANN is based on a feed forward multilayer perceptron, with a backpropagation algorithm, having 3 input variables, OA, LH, and PT, one hidden layer with sigmoidal function and one output layer with one neuron and linear function. The network has one output variable, roughness parameter Ra. The multilayer perceptron has been proven to be a universal approximator [27].

A database with 125 datasets was used. It was randomly divided into two groups: a first group of datasets for training and validation of the networks, and a second group of datasets to test the results, in order to assess the goodness of the selected network. In order to compare the behavior of the different networks, the Mean Absolute Error (MAE) of the test data, in µm, was used (Equation (1)).

(1) $$MAE=\frac{\sum \left|Objective\;output-Simulated\;output\right|}{Number\;of\;datasets}$$

where,

Objective output is the measured Ra value, in µm,

Simulated output is the Ra value provided by a network, in µm,

Number of datasets is the total number of datasets, in this case 125.

In order to assess the effect of the ANN parameters on MAE, different ANN models were obtained, in which three factors were varied:

1. Number of neurons in the hidden layer (4 levels).
2. Training algorithm used (3 levels).
3. Distribution of the dataset patterns among training, validation and test data (2 levels).

There are 24 sets of networks because this is the result of all combinations of 4 levels for the number of neurons (4, 5, 6 and 7), 3 levels for the training algorithm (LM, RB and SCG), and 2 levels for the distribution of datasets (70% train + 15% validation, 55% train + 30% validation). This is a full factorial design on the created networks (4 × 3 × 2 = 24).

Each factor of the networks is explained in detail in the next subsections.

#### 2.3.1. Number of Neurons in the Hidden Layer

In order to define the range of values for the number of neurons to be tested, the starting point was the work by Lawrence and Peterson [28] and Feng et al. [29], where they optimize the number of neurons in the hidden layer as the summation of the number of inputs and outputs. Thus, with the start point of 4 neurons in the hidden layer, corresponding to 3 inputs and 1 output, different number of neurons were tested (Table 2).

#### 2.3.2. Training Algorithm

A second factor that defines the networks is the training algorithm. Three different algorithms were selected and tested, which are usually employed in models with multilayer perceptron with backpropagation algorithm (Table 3).

#### 2.3.3. Distribution of Datasets

A third factor that configures the training process of the network is the distribution of datasets among the training, validation, and test datasets. According to different studies [30,31], and depending on the number of experiments performed and the degree of complexity of the problem, the following ranges are recommended:

Training data between 50 and 70%.

Validation data between 15 and 25%.

Test data between 15 and 25%.

In this study, the test data are used to compare the different networks. For this reason, the same test data are used in all cases. Thus, the percentage of test datasets is defined as 15%, and the rest of the datasets, 85%, are used to train and validate the networks, according to the following distributions of datasets or datasets splitting ratios (Table 4).

## 3. Results and Discussion

Table A1 in the Appendix A shows the experimental Ra values for the 125 3D printing experiments that were carried out.

### 3.1. Study About Different Networks

Using the experimental results for the 125 experiments, 24 different networks were tested, according to the definition in Section 2.3.1. The MAE values of the networks are presented in Table 5. The name of each network consists of the acronym of the training algorithm followed by the number of neurons and the dataset distribution. For instance, LM-4-7015 corresponds to the Levenberg–Marquardt algorithm, with four neurons in the hidden layer, and with 70–15% data splitting ratios.

**Figure 5:**
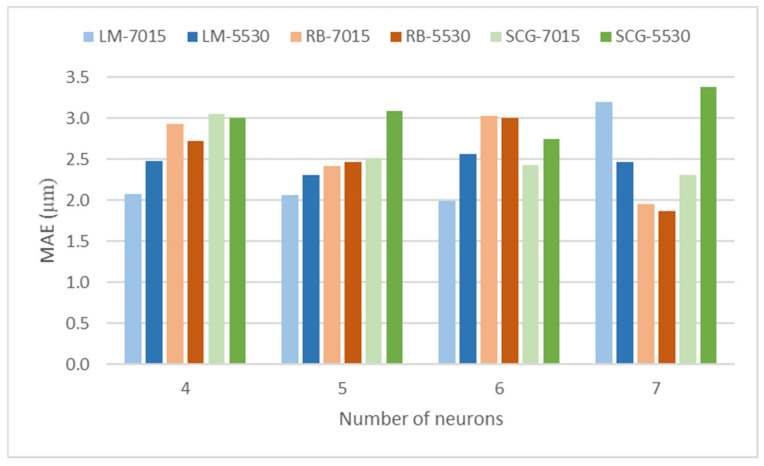
MAE values for the different network structures.

Figure 5 depicts the MAE values for the different combinations of training algorithm and dataset distribution, as a function of the number of neurons in the hidden layer.

Table 5 and Figure 5 show that, as a general trend, when four, five, or six neurons are considered, LM models provide lower MAE values than RB and SCG models. However, when seven neurons are used, RB models lead to lower values than the other models. Specifically, the best results are obtained with the RB algorithm, seven neurons and a distribution of 55% training data, 30% validation data, and 15% test data, with MAE of 1.874 µm (network 16). A similar number of neurons in the hidden layer, eight, was reported by Sood et al. [32] to model the compressive strength of parts in FFF processes, with a model having five input variables: layer thickness, orientation, raster angle, raster width, and air gap. Similarly, Saad et al. [33] selected eight neurons in each one of the two hidden layers of their model for roughness, with four input variables: layer thickness, print speed, print temperature, and outer shell speed. On the contrary, other authors used models with a higher number of neurons, for example 150, in a model with six input variables [24]. As for the algorithm, different options are available in FFF processes. For example, Mahapatra et al. used the Levenberg–Marquardt one [34], with the advantage of the faster training of ANN. Other authors like Monticeli et al. [35] employed the Resilient Backpropagation algorithm in order to predict the bending properties of FDM printed parts. Hosseini et al. [36] used the Scaled Conjugate Gradient backpropagation algorithm, together with the Levenberg–Marquardt algorithm and the Bayesian regularization in ANN combined with the genetic algorithm, in order to model the behavior of sinusoidal structures under quasi-static loading. They found that the Levenberg–Marquardt algorithm depended less on the number of neurons than the other algorithms did. In this work, LM models with four, five, and six neurons provided similar MAE values, while higher MAE values were obtained when seven neurons and the 7015 distribution were selected (network 7, LM-7-7015). On the other hand, increasing the number of training datasets does not always lead to lower error [36]. Similarly, in this work, in some cases using 70% of training datasets implies a higher error than using 55% of training datasets (for example, network 9, RB-4-7015, leads to higher error than network 10, RB-4-5530).

### 3.2. Optimization of Simulated Ra Values

In order to optimize roughness, the lowest simulated Ra values were searched among the 125 datasets. In Table 6, the lowest simulated Ra values for network 16 are presented.

The lowest simulated Ra values correspond to the low print orientation angle of 0° and the low layer height of 0.05 mm, with a high printing temperature equal or higher than 200 °C. Thus, in order to minimize surface roughness, a low orientation angle and a low layer height need to be selected, with a relatively high printing temperature.

### 3.3. Study About the Database Size

In the study, network number 3 was selected, LM-5-7015, with the LM learning algorithm, five neurons in the hidden layer and a structure of 70% training datasets, 15% validation datasets, and 15% test datasets. According to Table 5, it provided a MAE value for the test data of 2.068 μm, which is in the middle range for MAE.

The database size or total number of datasets is a key parameter for the configuration of neural networks [37]. In order to evaluate its influence on the performance of the networks, first, the 19 test datasets were separated from the 125 datasets. Then, four different configurations with different total number of datasets were selected, according to Table 7.

A is the original configuration, with 106 training datasets and a total amount of 125 datasets. Configurations B, C, and D correspond to 81, 56, and 31 training datasets, with a total amount of 100, 75, and 50 datasets, respectively.

The selected network was trained with the different number of training datasets that are defined in Table 7. In configurations B, C and D, the training was repeated three times, with a different random selection of the training datasets and the validation datasets.

Finally, the MAE values were calculated for each configuration. The results are presented in Table 8.

It is observed that, the lower the number of total datasets, the higher the average MAE of the networks. 

**Figure 6:**
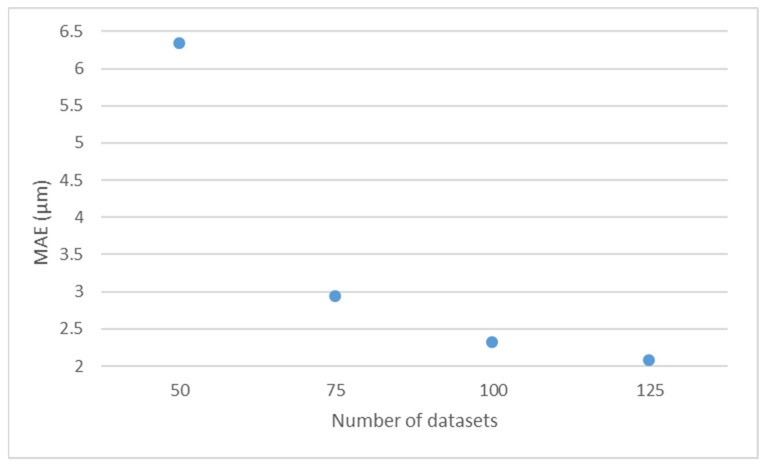
Average MAE values for the different number of datasets.

Figure 6 shows MAE as a function of total number of datasets. It is observed that, when the total number of datasets increases from 50 (configuration D) to 75 (configuration C), the average MAE of the network decreases substantially.

Thus, the higher the number of total datasets, the better the simulation is. Moreover, when the number of total datasets decreases to below 50, the network loses its effectivity.

## 4. Conclusions

Regarding the different neural network configuration studied, the main conclusions are as follows:

When a low number of neurons is selected (either four, or five), the LM algorithm provides better results than the other algorithms. However, lowest error among the networks studied corresponds to seven neurons, RB algorithm and 5530 distribution. According to this network, Ra values are minimized when a low orientation angle of 0°, a low layer height of 0.05 mm, and a relatively high printing temperature above 200 °C are selected.

The RB training algorithm is less sensitive to the relative variation of the train and validation data than the other algorithms tested, with similar results for 70% training datasets and for 55% training datasets. On the contrary, as a general trend, LM and SCG training algorithms behave better when 70% training datasets are selected, except for four neurons with the SCG algorithm and seven neurons with the LM algorithm.

Regarding the size of the total database, the lower the number of datasets, the higher the error of the network, with a sharp increase in MAE when 50 datasets are considered.

This work will help to select appropriate ANN parameters in order to reduce roughness in FFF processes. It will also contribute to the definition of efficient ANN models, which can be used in industry to optimize the 3D printing parameters from the experimental roughness values.

Although the main parameters in the ANN configuration have been considered in this study, future research directions could include other ANN parameters such as the type of functions employed. In addition, the same or similar prediction models could be tested to study other responses such as the dimensional accuracy of porosity of the 3D-printed parts.

## Figures and Tables

**Table 1:** **3D** printing parameters.

Parameter	Value
Nozzle diameter (mm)	0.4
Infill ratio (%)	80
Print speed (mm/s)	40
Print orientation angle (°) (OA)	0, 20, 40, 60, and 80
Layer height (mm) (LH)	0.05, 0.10, 0.15, 0.20, and 0.25
Printing temperature (°C) (PT)	190, 195, 200, 205, and 210

**Table 2:** Number of neurons in the hidden layer.

Number of Neurons in the Hidden Layer			
4	5	6	7

**Table 3:** Training algorithm.

Training Algorithm		
Levenberg–Marquardt (LM)	Resilient-Backpropagation (RB)	Scaled Conjugate Gradient (SCG)

**Table 4:** Distribution of datasets.

Distribution of Datasets	
70% train + 15% validation (7015)	55% train + 30% validation (5530)

**Table 5:** MAE values for the different networks.

Network Nr.	Network Name	MAE (µm)
1	LM-4-7015	2.075
2	LM-4-5530	2.477
3	LM-5-7015	2.068
4	LM-5-5530	2.309
5	LM-6-7015	1.997
6	LM-6-5530	2.569
7	LM-7-7015	3.203
8	LM-7-5530	2.470
9	RB-4-7015	2.926
10	RB-4-5530	2.718
11	RB-5-7015	2.417
12	RB-5-5530	2.463
13	RB-6-7015	3.024
14	RB-6-5530	3.009
15	RB-7-7015	1.953
16	RB-7-5530	1.874
17	SCG-4-7015	3.048
18	SCG-4-5530	3.006
19	SCG-5-7015	2.514
20	SCG-5-5530	3.088
21	SCG-6-7015	2.425
22	SCG-6-5530	2.749
23	SCG-7-7015	2.315
24	SCG-7-5530	3.381

**Table 6:** Lowest Ra values among all datasets, simulated with network 16.

Experiment	OA (°)	LH (mm)	PT (°C)	Simulated Ra (µm)
89	0	0.05	210	2.802
23	0	0.05	205	3.040
41	0	0.05	200	3.678

**Table 7:** Number of datasets for the different configurations.

Configuration	Total Datasets	Test Datasets	Training Datasets
A	125	19	106
B	100	19	81
C	75	19	56
D	50	19	31

**Table 8:** MAE values for each configuration.

Configuration	MAE (µm)	Average MAE (µm)
A	2.068	2.068
B_1	2.147	2.312
B_2	2.197	
B_3	2.594	
C_1	3.594	2.940
C_2	2.565	
C_3	2.661	
D_1	7.617	6.347
D_2	6.447	
D_3	4.977	

## Data Availability

The data presented in this study are available on request from the corresponding author.

## Appendix A

Table A1 contains the measured Ra values for the different experiments performed.

**Table A1:** Ra values for the 125 experiments.

Experiment	Layer Height (mm)	Temperature (°C)	Print Orientation Angle (°)	Ra (μm)
1	0.05	190	0	4.770
2	0.15	190	80	18.212
3	0.10	200	60	16.136
4	0.15	195	40	16.468
5	0.05	195	80	12.385
6	0.20	205	0	15.747
7	0.20	205	40	22.477
8	0.05	205	80	10.675
9	0.15	190	60	22.737
10	0.15	200	80	17.327
11	0.05	210	60	9.804
12	0.20	200	40	21.613
13	0.25	190	80	17.120
14	0.20	200	20	15.088
15	0.20	190	0	15.019
16	0.15	205	60	23.517
17	0.25	205	20	19.199
18	0.10	190	20	10.207
19	0.10	195	80	17.068
20	0.10	210	60	18.352
21	0.15	205	80	17.186
22	0.25	190	40	26.255
23	0.05	205	0	5.377
24	0.05	195	60	10.702
25	0.05	200	80	11.011
26	0.15	205	20	13.087
27	0.25	200	80	14.195
28	0.10	190	60	15.856
29	0.05	210	20	5.123
30	0.05	190	60	9.715
31	0.15	210	20	10.966
32	0.25	205	40	28.782
33	0.25	195	0	18.608
34	0.10	200	40	13.320
35	0.20	210	80	13.319
36	0.20	190	80	18.059
37	0.20	200	60	25.140
38	0.25	210	60	32.130
39	0.20	200	0	14.623
40	0.25	190	0	17.659
41	0.05	200	0	3.8395
42	0.10	200	80	15.957
43	0.15	210	0	10.404
44	0.25	195	40	29.163
45	0.25	210	80	17.306
46	0.20	195	0	14.640
47	0.20	210	20	15.477
48	0.25	210	0	17.429
49	0.10	205	80	14.565
50	0.05	190	80	10.966
51	0.05	210	40	9.816
52	0.25	195	80	14.261
53	0.10	195	0	6.968
54	0.15	210	60	24.996
55	0.20	190	20	15.450
56	0.15	205	0	10.067
57	0.15	200	40	18.815
58	0.25	210	40	28.472
59	0.20	205	80	15.486
60	0.15	205	40	18.014
61	0.15	210	80	15.073
62	0.15	210	40	18.563
63	0.15	195	80	15.829
64	0.20	195	60	30.767
65	0.10	205	20	9.166
66	0.05	210	80	11.414
67	0.05	205	60	9.971
68	0.25	190	20	21.956
69	0.20	195	80	21.739
70	0.10	205	0	7.154
71	0.25	210	20	19.904
72	0.25	200	60	30.778
73	0.20	195	40	26.123
74	0.10	210	40	12.475
75	0.05	190	40	8.538
76	0.10	200	20	8.093
77	0.10	210	20	8.470
78	0.10	195	40	12.415
79	0.15	190	40	24.313
80	0.25	195	20	30.005
81	0.15	195	0	10.074
82	0.05	205	40	11.703
83	0.20	205	60	24.597
84	0.10	190	40	16.937
85	0.05	200	60	9.490
86	0.25	205	80	28.090
87	0.05	200	20	7.186
88	0.25	195	60	48.851
89	0.05	210	0	3.880
90	0.20	205	20	21.630
91	0.25	200	20	27.833
92	0.10	190	0	6.609
93	0.10	190	80	21.895
94	0.20	210	60	29.740
95	0.20	200	80	32.232
96	0.05	195	40	9.922
97	0.15	190	20	18.600
98	0.10	210	0	6.792
99	0.15	195	60	33.355
100	0.25	205	60	45.503
101	0.25	205	0	18.356
102	0.05	195	0	4.252
103	0.20	210	40	36.138
104	0.15	200	20	16.747
105	0.15	200	60	25.378
106	0.20	190	40	33.423
107	0.15	200	0	11.104
108	0.05	200	40	7.987
109	0.20	210	0	14.775
110	0.05	195	20	6.834
111	0.20	190	60	40.021
112	0.10	205	60	24.393
113	0.10	205	40	18.439
114	0.05	205	20	7.736
115	0.15	195	20	11.570
116	0.15	190	0	9.994
117	0.25	200	0	17.590
118	0.10	195	20	8.327
119	0.10	200	0	6.645
120	0.10	210	80	13.600
121	0.20	195	20	14.002
122	0.05	190	20	7.576
123	0.25	200	40	30.875
124	0.10	195	60	16.863
125	0.25	190	60	55.106
